# Effect of the number and diversity of visual stimuli on the reproduction of short time intervals

**DOI:** 10.1177/03010066231190220

**Published:** 2023-08-15

**Authors:** Ali Bozorgmehr, Razieh Moayedi, Bahman Sadeghi, MohammadReza Molaei, Eli Brenner

**Affiliations:** 440827 Iran University of Medical Sciences (IUMS), Tehran, Iran; Payam Noor University of Tehran, Tehran, Iran; Institute of Biochemistry and Biophysics (IBB), 48425 University of Tehran, Tehran, Iran; 48511 Shahid Bahonar University of Kerman, Kerman, Iran; 1190 Vrije Universiteit Amsterdam, Amsterdam, The Netherlands

**Keywords:** time perception, timing, reproduction, tapping, variation, filled duration illusion

## Abstract

Presenting more items within a space makes the space look and feel bigger. Presenting more tones within a time interval makes the interval seem longer. Does presenting more *visual* items also make a time interval seem longer? Does it matter what these items are? A series of 2–4 images were presented sequentially on a screen. Participants had to press the spacebar to indicate either the interval between the first and the last item or the intervals between all items. The first and last items were red squares with onset asynchronies of 700, 900, or 1,100 ms. We found that the times between key presses were longer when additional items had different shapes and colors than when they were also red squares. With only red squares, the time may even decrease with the number of items. Whether one had to tap for all targets or only the first and the last hardly mattered.

Judgments of time are influenced by many factors. We have all experienced the fact that sometimes time appears to pass very slowly, such as when waiting for a train to arrive, whereas at other times it appears to pass very fast. There are many theories about how time is judged (Malapani & Fairhurst, 2002; Staddon, 2005; Tanaka & Yotsumoto, 2017), and how time is judged might depend on the scale involved: it might be different for sub-second judgments (Di Lernia et al., 2018; Droit-Volet et al., 2018) than for multiple-hour judgments (such as circadian rhythms; Hastings et al., 2018) or even longer durations. There is evidence for judgments of time being influenced by emotions (Gable et al., 2022), drugs (Steinberg, 1955), and diseases (Menegaldo et al., 2013; Tysk, 1984). In many such cases, one can measure the influence, but it is not clear why this particular influence is found. An example of a situation that might help us understand why judgments of time are affected by many factors is that there are many reports of time appearing to be longer when there are more events within that time (Nakajima et al., 1991; Repp, 2008), at least for intervals that last less than a few seconds (Ihle & Wilsoncroft, 1983) or even a fraction of a second (Zheng & Chen, 2020). This has mainly been studied in the context of sounds (and music). It is often considered to be specific to judgments of time, but actually, space appears to be larger when there are more items in it, both when looking at the space (Bulatov et al., 2021) and when feeling it (Sanders & Kappers, 2009). In touch, there is also an interesting distinction between an empty surface and no surface at all (Collier & Lawson, 2016). Thus, it might be a more general feature of magnitude estimation (Bueti & Walsh): incorrectly associating a number with length (in space or time).

Many studies have studied time perception using rhythmic tapping tasks, whereby participants were asked to tap in synchrony with a tone sequence and sometimes to continue tapping at the same rate when the tones stopped. When tapping along with the tones, participants typically tap too early (Repp, 2005). When the rate of the tones is tripled (Ono et al., 2022) and they are asked to tap along with every third tone, they still tap too early, but less so. This could be interpreted as evidence that adding a tone between the tones that one had to tap along with made the interval appear longer (Nakajima, 1987). In accordance with this, when continuing to tap after a rhythm stopped, the interval between taps was longer when there had been more tones between the ones indicating the rhythm (Repp, 2008). Repp (2008) also showed that the act of tapping itself makes a difference, although the difference is also present when judging intervals without tapping. The tendency to judge intervals to be longer when there were additional items within the interval was also observed when people were asked to tap at a higher rate than a previously indicated simple rhythm (Repp & Bruttomesso, 2010). In that case, they tapped faster, possibly to compensate for the interval appearing to be longer as a result of them themselves inserting additional taps. These studies all used auditory stimuli to define the temporal intervals. If the findings relate to general errors in magnitude estimation, there is no reason why they would not apply similarly to visually presented intervals. The main goal of the present study was therefore to determine whether we would also find that the time interval between two *visually* presented stimuli is judged to be longer if more stimuli are presented between them.

In addition to our main goal, we were interested to see whether the identity of the intervening items mattered, and whether their relevance to the task mattered. We considered that having more variation in the items might increase the judged duration, as might making the intervening items more relevant to the task. We conducted two experiments. In both experiments, participants first saw a temporal series of images and then indicated the timing of the presentation of the images by pressing the spacebar on a keyboard. In the first experiment, they pressed the spacebar twice to indicate the interval between the first and the last item that was presented. In the second experiment, they pressed the spacebar once for each item that was presented, in the rhythm of the presentation. They saw 2, 3, or 4 items. The first and the last were always red squares. The others could either also be red squares, or they could have different colors or shapes. In addition to examining whether the time appeared to be longer when there were more items between the first and the last tap, we also examined whether it appeared to be particularly long when the intervening items were different from the first and the last, and when one had to tap for each item rather than only for the first and the last. It is also conceivable that the time would appear to be shorter when intervening items were different than the first and the last, because for spatial vision the effect of having additional items between the items defining the distance that is judged was strongest when the additional items were identical to the ones that define the distance (Wackermann & Kastner, 2009).

## Methods

The experiments were developed using PsychoPy (Peirce, 2007). Participants sat in front of a computer screen and used the spacebar of the computer keyboard to indicate the relative timing of the stimuli that they had just seen. They could start responding at any time after the stimuli had been presented. The experiment was conducted using a Lenovo laptop with a 15.6-inch screen and a screen resolution of 1,920 × 1,080 pixels at 60 Hz. It was conducted in accordance with approval by the *Vaste Commissie Wetenschap en Ethiek van de Faculteit der Gedrags- en Bewegingswetenschappen*.

### Participants

Participants were selected on the basis of availability and willingness to take part in the study. They all provided informed consent. They all claimed to be physically and mentally healthy. Before starting the first experiments, the procedure was clearly explained to the participants. The two experiments were conducted in a quiet room. Each participant took part in both experiments, with a short break between the two. The whole procedure took approximately 45 min. In total, 27 individuals participated in the study. Two participants were later excluded because they did not have any trials in which they tapped 3 or 4 times when they should have (trials were excluded if participants did not tap the correct number of times). Consequently, the results are based on the data of 25 participants (9 women; 16 men; mean age: 31 years).

### Procedure

**Figure 1.**
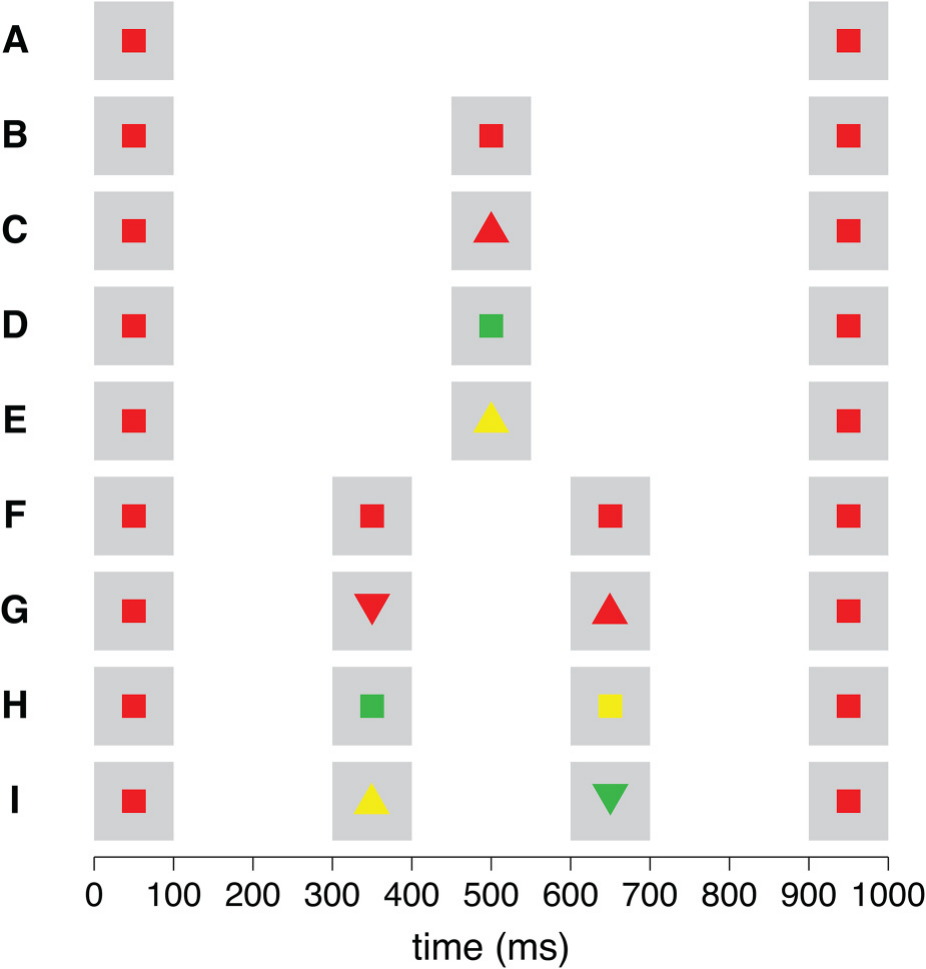
The nine combinations of stimuli for each time interval. Each presentation started and ended with a red square being presented for 100 ms. The last red square could appear at 700 ms, 900 ms (as shown here), or 1,100 ms after the first one appeared. These two items could be presented alone (A), or else either one (B–E) or two (F–I) items could be presented between them, at regular intervals. When there were additional items between the first and the last, such items could also be red squares (B, F) or they could be different: red triangles (C, G), yellow and/or green squares (D, H), or yellow and/or green triangles (E, I). The triangles could be oriented upwards or downwards. Their orientation and whether nonred items were yellow or green were determined at random.

The stimulus presentation always started and ended with a red square appearing at the center of the screen. The time interval between the onset of the first and the last red square was 700, 900, or 1,100 ms. In the time interval between the first and the last red squares, either no other item, one other item (a red, green, or yellow square or triangle), or two other items (two red, green, or yellow squares or triangles) were presented (Figure 1). The duration of presentation of each item was 100 ms and the time intervals between consecutive items were constant within each trial. We had 27 different types of trials, each of which was repeated 3 times. As a result, each experiment consisted of 81 trials.

The stimuli were the same in the two experiments. In the first experiment, participants were asked to reproduce the time interval between the first red square and the last red square. After each presentation, they had to press the space key twice, with the interval between the key presses being equal to the time between the presentation of the first and the last items (always red squares). Pressing the space key provided a short beep to confirm that the key press had been registered. In the second experiment, participants had to press the space key once for each item that they saw. Thus, when two additional items were presented between the first and the last, participants had to press the space key 4 times with the time intervals being equal to the time intervals between the presented stimuli.

### Analysis

We determined the time between the first and the last key-press for each trial. Trials were excluded if the number of key presses was incorrect. The error in reproducing the time between the first and the last presented item was determined by subtracting the time interval between presentation onsets from the time between the first and the last key-press. Thus, more positive values indicate that participants’ key presses were further apart in time than the interval that had been presented. The median timing error was determined for each experiment (tap for the first and the last, or for all items), time interval between the onset of the first and the last items (700, 900, or 1,100 ms), number of items (2, 3, or 4), and type of items (all red squares, or other items between the first and the last). Using the median avoids having to worry about the distribution and about outliers. Since there was no distinction between types of items when there were only two items, we used a linear mixed effects model (lme4 library of R) to evaluate whether the four factors (experiment, time interval, number of items, and type of item) or any of their interactions were significant. The model included the participant as a random variable: median value ∼ time interval × factor(experiment) × number of items × factor(type of item) + (1|participant).

## Results

**Figure 2.**
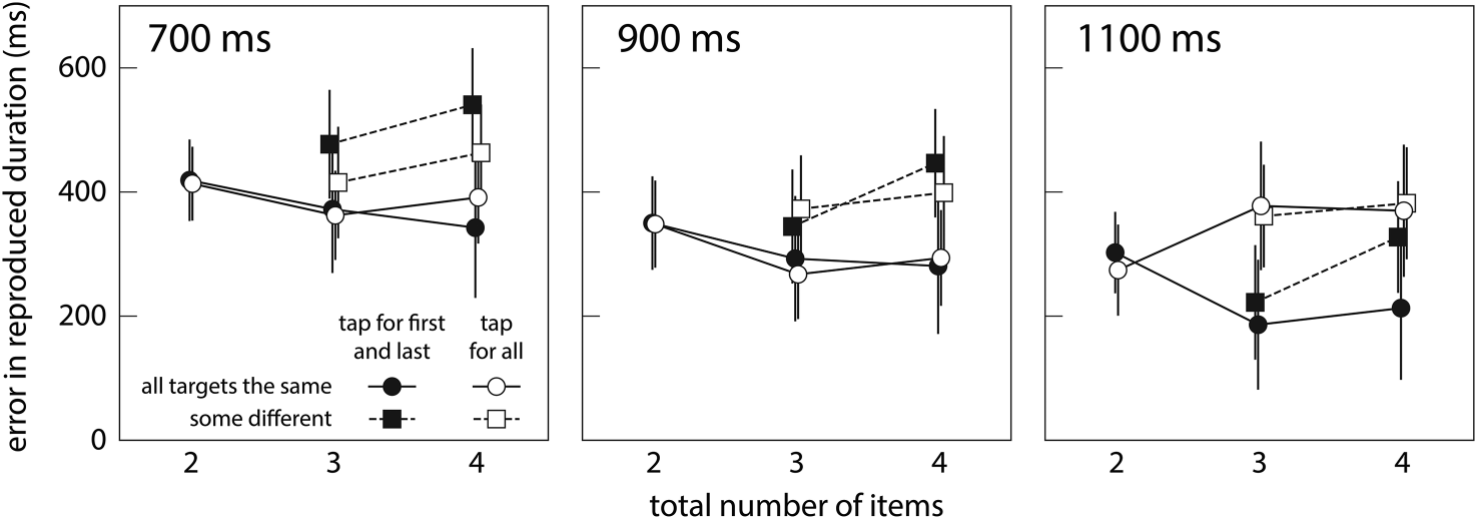
Error in the time between the first and the last tap as a function of the number of items. Positive values indicate that the duration between the first and the last tap was too long. Mean and standard error across participants’ median values. Data for the different time intervals between the onsets of the first and the last item are shown in separate panels. Solid symbols: Experiment 1. Open symbols: Experiment 2. Circles: All items are red squares. Squares: Items between the first and the last are not red squares.

The time between the first and the last tap was consistently too long (Figure 2). This was particularly so for shorter intervals: points for the 700 ms interval (left panel) are generally higher than those for the 1,100 ms interval (right panel). The linear mixed effects model confirmed this effect of the time interval between the first and the last stimulus, *F*(1,623) = 21.6, *p* < .001. More importantly, the time between the first and the last tap was generally longer when there were different kinds of items than when all items were red squares (squares higher than circles in Figure 2; *F*(1,623) = 10.2, *p* = .001). The time between the first and the last tap also appears to decrease with the number of items when all targets were red squares (circles in Figure 2) but to increase with the number of items when there were different kinds of items (squares in Figure 2), resulting in a significant interaction between the kind of item and the number of items, *F*(1,623) = 4.62, *p* = .032. Finally, for the longest time interval, the time between the first and the last tap appears to be longer when tapping for all targets (open symbols; Experiment 2) than when only tapping for the first and the last (solid symbols; Experiment 1), giving rise to a significant interaction between the experiment and the time interval, *F*(1,623) = 5.93, *p* = .015. None of the other main effects (experiment; number of items) or interactions were significant.

## Discussion

The main goal of the present study was to determine whether the time interval between two visually presented stimuli is judged to be longer if more stimuli are presented between them. The answer to this question is more complicated than we had expected: it might be the case when the intervening stimuli are different than the first and the last, but it is certainly not the case when the items all look the same (contrary to findings for spatial vision; Wackermann & Kastner, 2009). Presenting tones that sound the same between the ones that define an interval does make the time between the first and the last tone appear to be longer (Horr & Di Luca, 2015; Zheng & Chen, 2020), so in this respect judging the time interval between visual stimuli may not be the same as judging the time interval between auditory stimuli. Consequently, our findings do not support the idea that the duration of time intervals is simply influenced by incorrectly associating numbers with length.

We did find some support for the idea that having more variation in the items increases the judged duration (squares above circles in Figure 2). Each item was only presented for 100 ms in our study, so it is unlikely that overestimating the durations of the presentations of the less common items themselves is responsible for the duration of sequences with more variation in the items appearing to be longer (Tse et al., 2004). It is more likely that having seen more different items gave participants the impression that more time had passed between the presentations, possibly mediated by encountering observations that did not match one's expectations (Matthews & Gheorghiu, 2016), even if the aspects in which the items differed (color, shape, and orientation) were not relevant to the task. More generally, our study supports previous findings with auditory stimuli showing that *how* the time between the relevant items is filled makes a difference, not only *whether* it is filled (Horr & Di Luca, 2015).

Making the intervening items themselves more relevant by tapping for each of them did not increase the time between the first and the last tap systematically, but it did increase the time between the first and the last tap for the longest duration. If it had only been the case for the shortest duration we could have attributed it to limitations in how fast one can move: having to tap 4 times within 700 ms is quite challenging. But, why it would be the case for the longest duration is not obvious to us. It might be related to the stimulus rather than the response. When the time interval between two visual stimuli is short it can be difficult to perceive those stimuli separately (Hoerl et al., 2020). That might be why participants occasionally, and the two excluded participants even often, misjudged the number of stimuli (or at least tapped a wrong number of times). Maybe there is a transition as the stimuli become more distinct between tapping a number of times during a certain interval, in which case the tapping mode does not matter, and tapping separately for each item when asked to do so.

We had not anticipated the clearest effects that we found: the tendency for the interval between the first and the last tap to be too long, and this tendency being strongest for the shortest interval between the first and the last item. The latter finding might be caused by an overall bias towards the mean (response) duration, which would increase the value for the shortest interval (700 ms) and decrease it for the longest (1,100 ms). This tendency also appeared to be present in comparable conditions to our 2-item data in earlier studies, both when the items were flashed on a screen (Mitsudo et al., 2012), as in our study, and when they were sounds (Bratzke et al., 2017). However, those studies did not find an overall tendency for the interval between the taps to be too long. Adding sounds between the flashes did increase the interval, leading to a tendency for the interval between taps to be too long (Mitsudo et al., 2012). In that study, the time interval between the flashes, which is what participants were asked to reproduce, was 600 or 800 ms. Looking at the most similar condition of the present study we see a similar increase in the reproduced interval when different items are presented between the items specifying the interval, but the overall tendency for the interval between the taps to be too long was much larger. The extent of the over-estimation in our study is more than the 100 ms target presentation, so it cannot simply be explained by participants tapping to match the onset of the first and offset of the last stimulus. We, therefore, have no explanation for this finding.

A clear limitation of our study is the low number of trials in some cases. We kept the number low to ensure that participants could perform both tasks within about an hour. To increase variability in the items without making the presence of items other than the red squares too surprising, we included more combinations of stimuli containing different items than combinations with only red squares (Figure 1). Consequently, the results when there were only red squares (circles in Figure 2) are based on three trials per participant, while the results when there were also other items (squares in Figure 2) are based on nine trials per participant. The fact that we found quite similar results for the 3-time intervals is reassuring, but both the low number of trials and the fact that there was always much too much time between the first tap and the last, raise some doubts as to whether this is the best method for studying how having additional visual items influences the perceived time between events.

In conclusion, we do not find that showing more items sequentially within a given time period makes that time interval appear longer. However, showing more different items might. Finally, whether one is required to tap along with each item or only to match the time between the first and the last item does not appear to matter when the items are all presented within a brief interval, but there is some suggestion that this might change as the total time interval exceeds 1s.
